# Genetic Modification of *Sodalis* Species by DNA Transduction

**DOI:** 10.1128/mSphere.01331-20

**Published:** 2021-02-17

**Authors:** Chelsea M. Keller, Christopher G. Kendra, Roberto E. Bruna, David Craft, Mauricio H. Pontes

**Affiliations:** a Department of Pathology and Laboratory Medicine, Pennsylvania State University College of Medicine, Hershey, Pennsylvania, USA; b Department of Microbiology and Immunology, Pennsylvania State University College of Medicine, Hershey, Pennsylvania, USA; Clemson University

**Keywords:** *Sodalis praecaptivus*, *Sodalis glossinidius*, insect endosymbiont, symbiont, transformation, transduction, genetic modification, plasmid transfer, transposition, bacteriophage P1, gene disruption, mutation, paratransgenesis, bacteriophage transduction, symbiosis

## Abstract

Bacteriophages (phages) are ubiquitous in nature. These viruses play a number of central roles in microbial ecology and evolution by, for instance, promoting horizontal gene transfer (HGT) among bacterial species. The ability of phages to mediate HGT through transduction has been widely exploited as an experimental tool for the genetic study of bacteria. As such, bacteriophage P1 represents a prototypical generalized transducing phage with a broad host range that has been extensively employed in the genetic manipulation of Escherichia coli and a number of other model bacterial species. Here we demonstrate that P1 is capable of infecting, lysogenizing, and promoting transduction in members of the bacterial genus *Sodalis*, including the maternally inherited insect endosymbiont Sodalis glossinidius. While establishing new tools for the genetic study of these bacterial species, our results suggest that P1 may be used to deliver DNA to many Gram-negative endosymbionts in their insect host, thereby circumventing a culturing requirement to genetically manipulate these organisms.

**IMPORTANCE** A large number of economically important insects maintain intimate associations with maternally inherited endosymbiotic bacteria. Due to the inherent nature of these associations, insect endosymbionts cannot be usually isolated in pure culture or genetically manipulated. Here we use a broad-host-range bacteriophage to deliver exogenous DNA to an insect endosymbiont and a closely related free-living species. Our results suggest that broad-host-range bacteriophages can be used to genetically alter insect endosymbionts in their insect host and, as a result, bypass a culturing requirement to genetically alter these bacteria.

## INTRODUCTION

Bacteriophages (phages) are the most abundant and diverse biological entities on the planet. With an estimated population size greater than 1 × 10^31^ (1), these bacterial viruses play essential ecological and evolutionary functions. Phages control the size of bacterial populations and shape the diversity of microbial communities by modulating the abundance of bacterial lineages and promoting, directly and indirectly, the exchange of genetic information among species (2, 3). Historically, phages have played a central role in the development of molecular biology, enabling, for instance, the establishment of DNA as the genetic material of living cells (4). Today, phages are widely used as tools in the study of bacteria. For instance, generalized transducing phages such as P1 allow the rapid transfer of DNA among bacterial strains, greatly facilitating genetic dissection of biological processes (5).

P1 is a temperate bacteriophage capable of alternating between lytic and lysogenic infection. P1 was initially described in studies involving lysogenic strains of Escherichia coli (6). This phage is capable of mediating generalized transduction (7), a property that has fostered its adoption as an important experimental tool for the genetic analysis and manipulation of E. coli (5, 8). Notably, in addition to its habitual E. coli host, P1 can also infect a large number of Gram-negative bacterial species (8–12). This broad host range, along with its well-characterized molecular biology and established experimental procedures, has prompted the use of this phage as an experimental tool for the delivery of DNA to a large number of bacterial species (13–18). Here we establish that P1 is capable of infecting two members of the bacterial genus *Sodalis*, including Sodalis glossinidius (19, 20).

Sodalis glossinidius is a maternally inherited, Gram-negative bacterial endosymbiont of tsetse flies (*Glossina* spp.; Diptera: *Glossinidae*). Similar to other insect endosymbionts, S. glossinidius exists in a stable, chronic association with its insect host and undergoes a predominantly maternal mode of transmission (21–23). Notably, like other insect endosymbionts, this bacterium has undergone an extensive process of genome degeneration as a result of a recent ecological transition from free-living existence to permanent host association (24, 25). Because this process is accompanied by the loss of metabolic capability and stress response pathways (24–29), *S. glossinidius* has proven refractory to harsh artificial DNA transformation procedures that are commonly employed in model organisms such as Escherichia coli (29). Consequently, this bacterium has remained genetically intractable (30).

**FIG 1:**
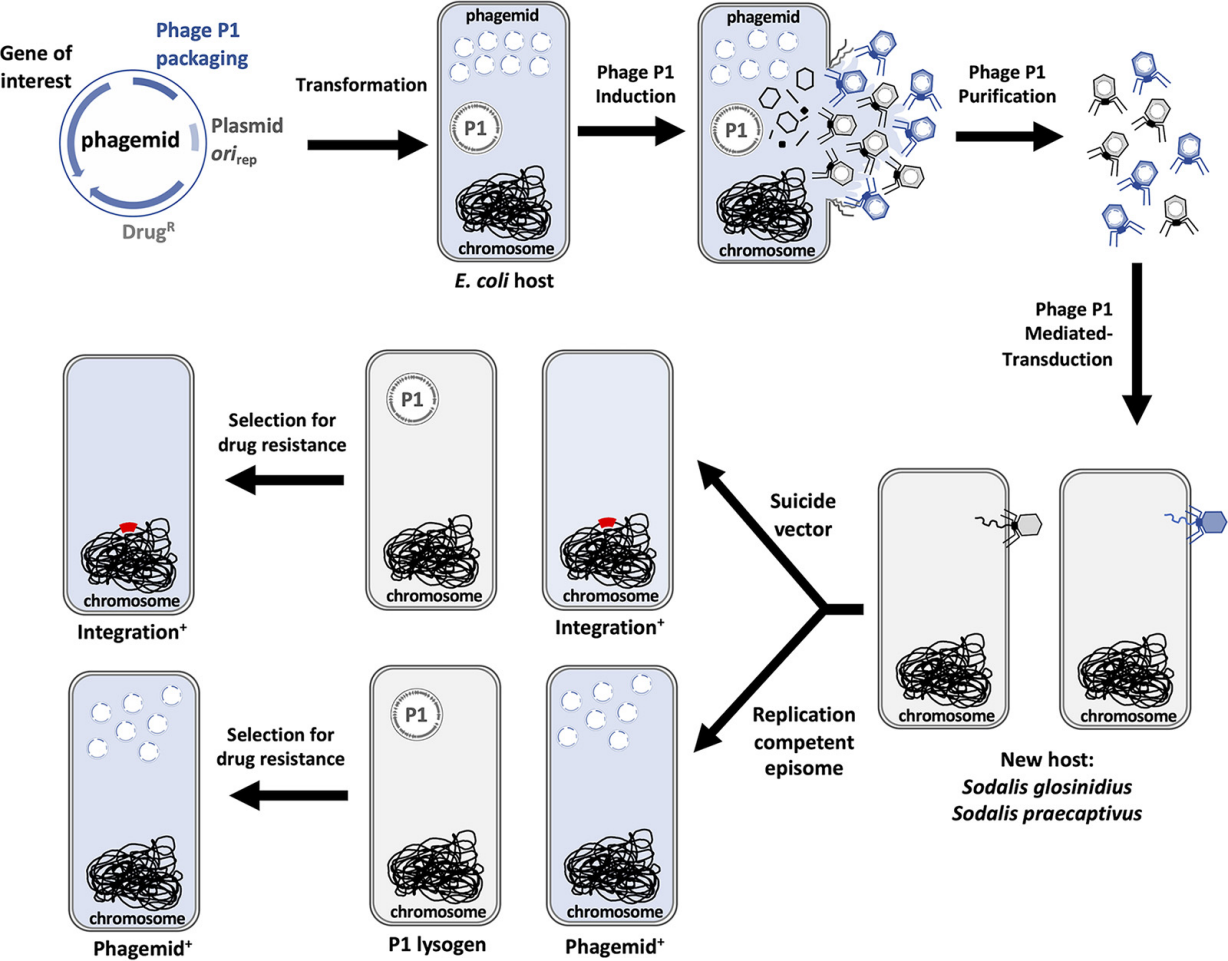
Cartoon representation depicting a workflow of the transduction procedure developed for introduction of phagemids in *Sodalis* species. Following the direction of the arrows, an E. coli P1 lysogen host is transformed with a P1 phagemid. The phagemid is packaged following induction of the P1 prophage, and lysates derived from culture supernatant are used to infect a *Sodalis* recipient strain. Cells receiving the phagemid are subsequently isolated on plates containing a selective agent.

In this study, we demonstrate that the bacteriophage P1 is capable of infecting, lysogenizing, and promoting transduction in Sodalis glossinidius, and its free-living close relative, the plant-associated and opportunistic pathogen Sodalis praecaptivus (19, 20). We demonstrate that P1 can be used to mediate generalized transduction of chromosomal and extrachromosomal DNA in S. praecaptivus. We use P1 to transduce autonomous replicating phagemids containing an array of reporter genes and Tn*7* transposition systems harboring fluorescent proteins for chromosomal tagging. Finally, we developed a suicide phagemid containing a mariner transposase for random mutagenesis of bacterial strains susceptible to P1 infection. This study establishes a new efficient method for genetic manipulation of *Sodalis* species (Fig. 1) that can be readily adapted to other Gram-negative bacteria. Furthermore, these results provide a potential means for the genetic modification of bacterial endosymbionts, in their insect host, through the use of P1 as a DNA delivery system.

## RESULTS

### Bacteriophage P1 infects, lysogenizes, and forms phage particles in Sodalis glossinidius and Sodalis praecaptivus.

**FIG 2:**
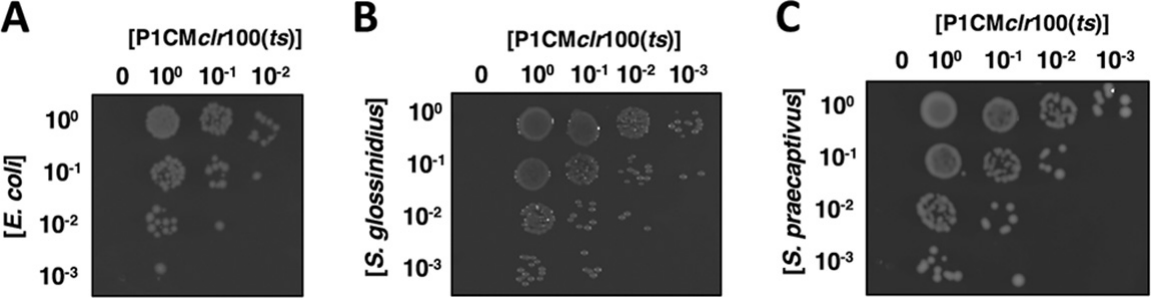
Infection of bacterial strains by phage P1. Lysates derived from an E. coli P1CM*clr*-100(ts) lysogen (KL463) were used to infect E. coli MG1655 (A), Sodalis glossinidius (B), and Sodalis praecaptivus (C). Plates depict the formation of chloramphenicol-resistant colonies as functions of the concentration of bacteria (vertical axis) and the concentration of P1CM*clr*-100(ts) lysates (horizontal axis). Note that P1 infection conditions for the strains are different (see Materials and Methods), and images do not reflect efficiency of P1 infection. Images show representative plates of at least three routine experiments.

P1CM*clr*-100(ts) is a thermo-inducible P1 variant harboring a chloramphenicol-resistant marker. P1CM*clr*-100(ts) forms chloramphenicol-resistant lysogens at low temperatures (≤30°C) but produces phage particles at higher temperatures (≥37°C) (31). Consequently, infection of E. coli by P1CM*clr*-100(ts) yields chloramphenicol-resistant lysogens at 30°C. We took advantage of these P1CM*clr*-100(ts) properties to test whether S. glossinidius and S. praecaptivus were susceptible to P1 infection. We exposed cultures of these bacteria to increasing concentrations of P1CM*clr*-100(ts) phage particles and subsequently plated dilutions on solid medium containing chloramphenicol. We established that, similar to the E. coli control (Fig. 2A), exposure to increasing concentrations of P1CM*clr*-100(ts) particles yielded increasing numbers of chloramphenicol-resistant colonies in both *S. glossinidius* (Fig. 2B) and *S. praecaptivus* (Fig. 2C). Importantly, no chloramphenicol-resistant colonies were observed in cultures that were not exposed to P1CM*clr*-100(ts) particles (Fig. 2B and C).

**FIG 3:**
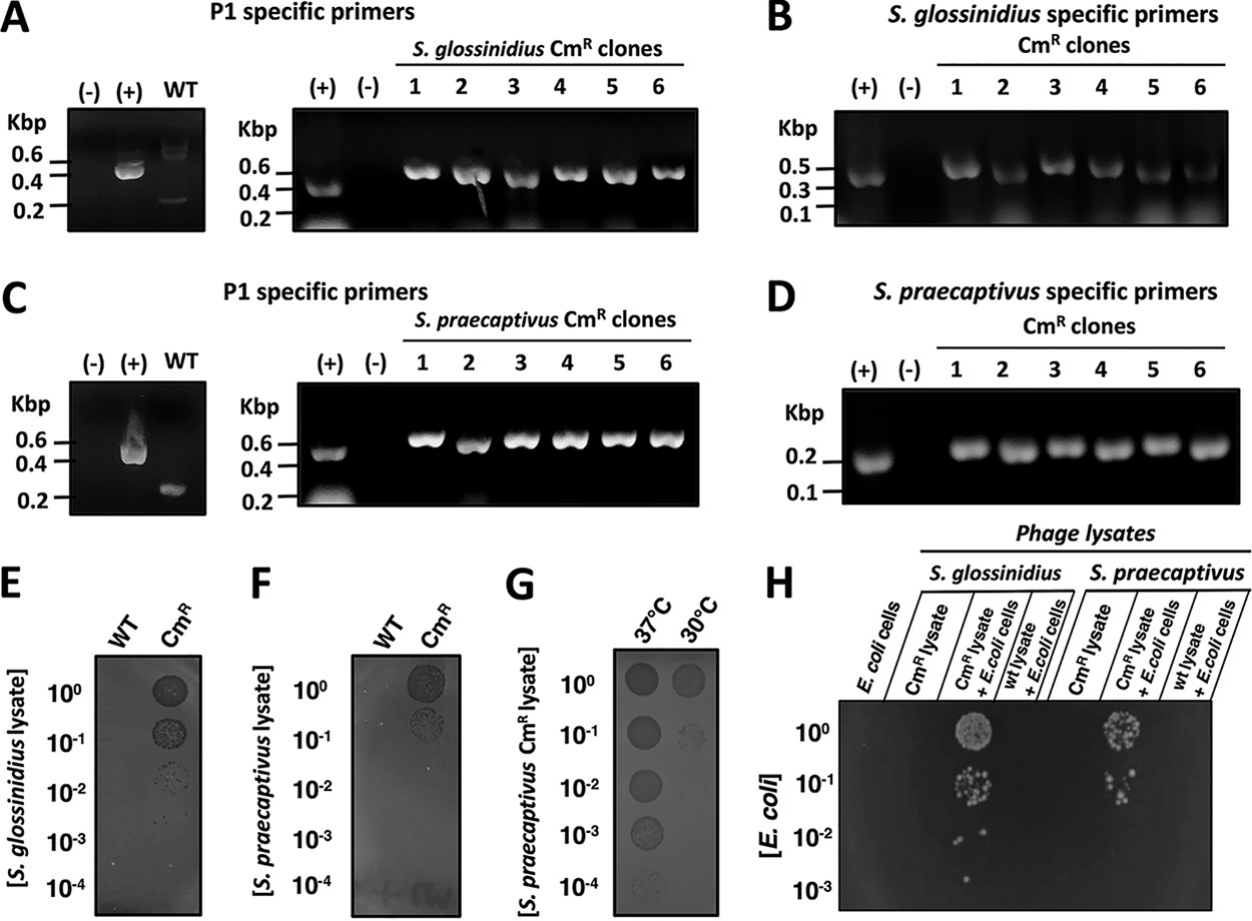
Lysogenization and production of infective phage particles by Sodalis glossinidius and Sodalis praecaptivus P1 lysogens. (A) Detection of P1 *pacB* gene by PCR and agarose gel electrophoresis in *S. glossinidius* chloramphenicol-resistant clones that emerged following exposure to an E. coli P1CM*clr*-100(ts) lysogen (KL463). (B) Detection of an *S. glossinidius*-specific DNA fragment in clones depicted in panel A by PCR and agarose gel electrophoresis. (C) Detection of the P1 *pacB* gene by PCR and agarose gel electrophoresis in *S. praecaptivus* chloramphenicol-resistant clones that emerged following exposure to an E. coli P1CM*clr*-100(ts) lysogen (KL463). (D) Detection of an *S. praecaptivus-*specific DNA fragment in clones depicted in panel C by PCR and agarose gel electrophoresis. WT, wild type; (−), no DNA control; (+), positive control. (E to G) Formation of phage plaques on soft agar embedded with E. coli MG1655. Soft agar plates were spotted with dilutions of lysates derived from wild-type and *S. glossinidius* chloramphenicol-resistant (Cm^r^) P1CM*clr*-100(ts) lysogen (MP1705) (E), wild-type and *S. praecaptivus* Cm^r^ P1CM*clr*-100(ts) lysogen (MP1703) (F), and *S. praecaptivus* Cm^r^ P1CM*clr*-100(ts) lysogen (MP1703) grown either at 37°C or 30°C. Plates are representative of the plates of routine experiments. (H) Emergence of Cm^r^ E. coli MG1655 following exposure to lysates derived from wild-type *S. glossinidius*, *S. glossinidius* Cm^r^ P1CM*clr*-100(ts) lysogen (MP1705), wild-type *S. praecaptivus*, and *S. praecaptivus* Cm^r^ P1CM*clr*-100(ts) lysogen (MP1703). The plate is representative of the plates of at least three experiments.

That these colonies were P1 lysogens, as opposed to recombinants harboring only the P1-derived chloramphenicol-resistant marker, was supported by several lines of evidence. First, the presence of a P1 DNA fragment was detected by polymerase chain reaction (PCR) in both chloramphenicol-resistant *S. glossinidius* (Fig. 3A and B) and *S. praecaptivus* clones (Fig. 3C and D), but not in the wild-type strains (Fig. 3A to D, left side). This indicated that chloramphenicol-resistant cells harbor at least part of the P1CM*clr*-100(ts) genome. Second, lysates prepared from *S. glossinidius* and *S. praecaptivus* chloramphenicol-resistant clones, but not their wild-type counterparts, formed plaques in soft agar cultures of E. coli grown at 37°C, a temperature that induces P1CM*clr*-100(ts) lytic replication (31) (Fig. 3E and F). This indicated that chloramphenicol-resistant *S. glossinidius* and *S. praecaptivus* clones can produce phage particles that are lytic to E. coli grown at 37°C. Third, the lytic activity of lysates derived from chloramphenicol-resistant *S. praecaptivus* cultures propagated at 37°C was 10,000 times higher than those maintained at 30°C (Fig. 3G). This established that higher titers of phage particles were being produced in *S. praecaptivus* chloramphenicol-resistant clones at a temperature where P1CM*clr*-100(ts) becomes lytic. Finally, lysates derived from *S. glossinidius* and *S. praecaptivus* chloramphenicol-resistant clones, but not their wild-type isogenic counterparts, promoted the formation of chloramphenicol-resistant E. coli cells at 30°C (Fig. 3H). This indicated that the chloramphenicol-resistant marker can be transduced from *S. glossinidius* and *S. praecaptivus* back to E. coli.

In *S. glossinidius*, the frequency of chloramphenicol-resistant colonies arising following P1CM*clr*-100(ts) exposure was similar to those observed for the E. coli control cells, indicating that P1 infection occurs efficiently in this bacterium. In contrast, chloramphenicol-resistant *S. praecaptivus* colonies emerged at a lower frequency, and higher concentrations of bacterial cells were typically used in P1 infection experiments (see Materials and Methods). Notably, in P1-resistant Salmonella enterica, the efficiency of P1 infection can be drastically increased by mutations in either *galU* or *galE* (10). Because these mutations remove the O antigen by truncating the core region of the lipopolysaccharide (LPS) (32, 33), they presumably facilitate access of P1 to its host receptor—conserved structural motifs within the LPS core (8, 10). In particular, while the LPS of *S. praecaptivus* contains structural components attached to its core region, *S. glossinidius* is devoid of such structures (see Fig. S1A in the supplemental material). Nonetheless, the lower infectivity of P1 does not appear to be related to the physical occlusion of the P1 receptor by components present in the outer portion of the *S. praecaptivus* LPS. This is because a mutation in *galU* results in a truncated LPS in *S. praecaptivus* but does not affect P1 infectivity (Fig. S1). Hence, unlike S. enterica, this phenotype is not due to the presence of a P1-antagonizing structure(s) in the outer portion of *S. praecaptivus* LPS. Taken together, these results indicate that phage P1 is capable of infecting and lysogenize in *S. glossinidius* and *S. praecaptivus*.

10.1128/mSphere.01331-20.1 FIG S1 Effect of a *galU* mutation on LPS composition and P1 infectivity in *S. praecaptivus*. (A) Schematic representation depicting the inner membrane, peptidoglycan, and outer membrane of a prototypical Gram-negative bacterium. The inset shows a more detailed schematic of structural components of the LPS (left-hand side). Silver-stained sodium dodecyl sulfate-polyacrylamide gel electrophoresis (SDS-PAGE) of LPS purified from *S. praecaptivus galU* (CMK36), wild-type *S. praecaptivus*, and wild-type *S. glossinidius* (right-hand side). (B) Plates depicting the appearance of chloramphenicol-resistant colonies as functions of the concentration of bacteria (vertical axis) and the concentration of P1CM*clr*-100(ts) lysates (horizontal axis). Cm^r^ colonies emerge at similar frequencies in wild-type *S. praecaptivus* (left-hand side plate) and *S. praecaptivus galU* (CMK36) (right-hand side plate) when cells are exposed to equal amounts of P1CM*clr*-100(ts) particles. Download FIG S1, TIF file, 2.4 MB. Copyright © 2021 Keller et al. 2021 Keller et al. https://creativecommons.org/licenses/by/4.0/ This content is distributed under the terms of the Creative Commons Attribution 4.0 International license.

### P1 generalized transduction in S. praecaptivus.

**FIG 4:**
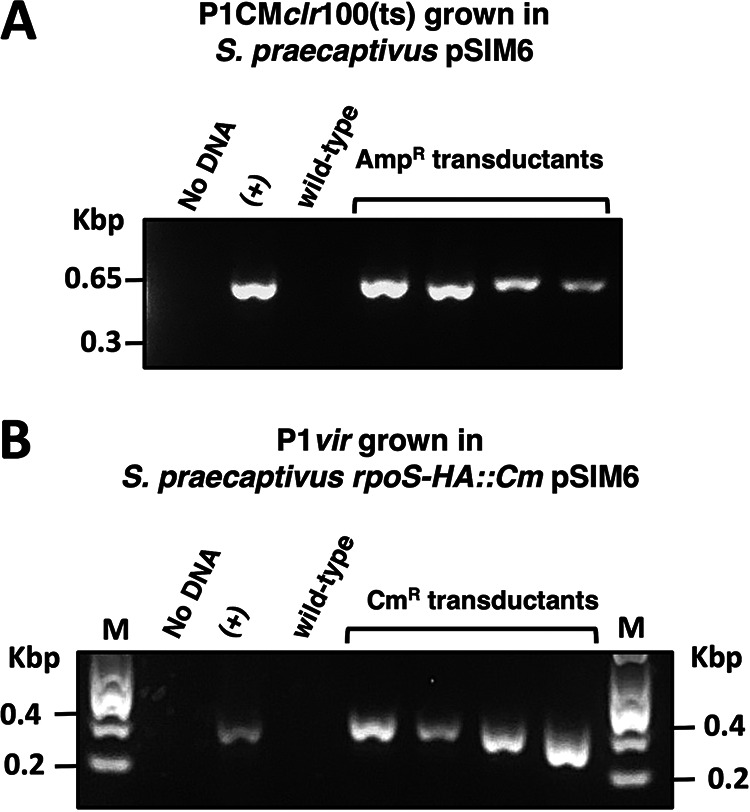
Bacteriophage P1-mediated generalized transduction in *S. praecaptivus*. (A) Detection of a pSIM6 DNA region by PCR and agarose gel electrophoresis in ampicillin-resistant (Amp^r^) *S. praecaptivus* transductants following exposure to lysates derived from *S. praecaptivus* Cm^r^ P1CM*clr*-100(ts) (MP1703) harboring Amp^r^ plasmid pSIM6. (B) Detection of the *rpoS-HA*::*Cm* chromosomal insertion by PCR and agarose gel electrophoresis in *S. praecaptivus* transductants following exposure to lysates of P1*vir* grown in *S. praecaptivus rpoS-HA*::*Cm* pSIM6 (MP1522) strain.

During the formation of P1 virions, approximately 0.05 to 0.5% of infective phage particles package random DNA fragments derived from the bacterial host (34). These particles can mediate the transfer of bacterial DNA across P1-susceptible strains through generalized transduction. In the laboratory, generalized transduction of DNA can be identified by virtue of genetic markers that are packaged in these phage particles and transferred between bacterial strains. Accordingly, we sought to determine whether P1 could mediate generalized transduction in *S. praecaptivus*. First, we exposed wild-type *S. praecaptivus* to phage lysates derived from an *S. praecaptivus* P1CM*clr*-100(ts) lysogen harboring the ampicillin-resistant (Amp^r^) plasmid pSIM6 (35). Following lysate exposure, we were able to retrieve Amp^r^ *S. praecaptivus* transductants. Importantly, Amp^r^ cells were absent from both phage lysates alone and cultures of wild-type *S. praecaptivus* that were not exposed to phage (data not shown). In agreement with the notion that these Amp^r^ clones were P1 transductants, diagnostic PCR revealed the presence of a pSIM6 fragment in these cells (Fig. 4A).

Next, we attempted to transduce a chromosomal chloramphenicol-resistant marker (*rpoS-HA*::*Cm*) using the P1 lytic strain P1*vir*. (This P1 strain is widely used as a transducing agent in E. coli due to its inability to lysogenize cells upon infection and the ease with which transducing lysates can be generated [5, 34].) We infected wild-type *S. praecaptivus* cells with P1*vir* lysates grown in an *S. praecaptivus rpoS-HA*::*Cm* pSIM6 strain. Whereas chloramphenicol-resistant (Cm^r^) cells emerged from wild-type *S. praecaptivus* exposed to phage, no Cm^r^ cells were obtained from phage lysates or cultures of naive wild-type *S. praecaptivus* alone (data not shown). Notably, diagnostic PCR indicated that chloramphenicol-resistant clones were transductants harboring the *rpoS-HA*::*Cm* genetic modification (Fig. 4B). Importantly, all of these *rpoS-HA*::*Cm* transductants were sensitive to ampicillin, indicating that P1*vir* mediated the transduction of a discrete portion of the *S. praecaptivus* genome. Taken together, these results indicate that bacteriophage P1 can be used to mediate generalized transduction in *S. praecaptivus*.

### Introduction of exogenous DNA in S. glossinidius and S. praecaptivus by P1-mediated guided transduction.

Whereas up to 0.5% of P1 particles can contain random fragments of bacterial host DNA (34), the vast majority of virions harbor P1 DNA. This is because the packaging of P1 genome into phage particles is guided by elements encoded within its DNA sequence (36, 37). Particularly, this packaging element can be cloned into plasmids (to produce phagemids) or incorporated into the bacterial chromosome to increase the frequency of P1-mediated transduction of adjacent DNA (17, 38, 39). Indeed, the P1 packaging element can increase the transduction of linked DNA by 1,600-fold above the levels obtained in generalized transduction (38). Hence, this DNA element can be used to increase the number of transducing particles and, consequently, the efficiency of DNA transfer among bacterial strains that are susceptible to P1 infection.

**FIG 5:**
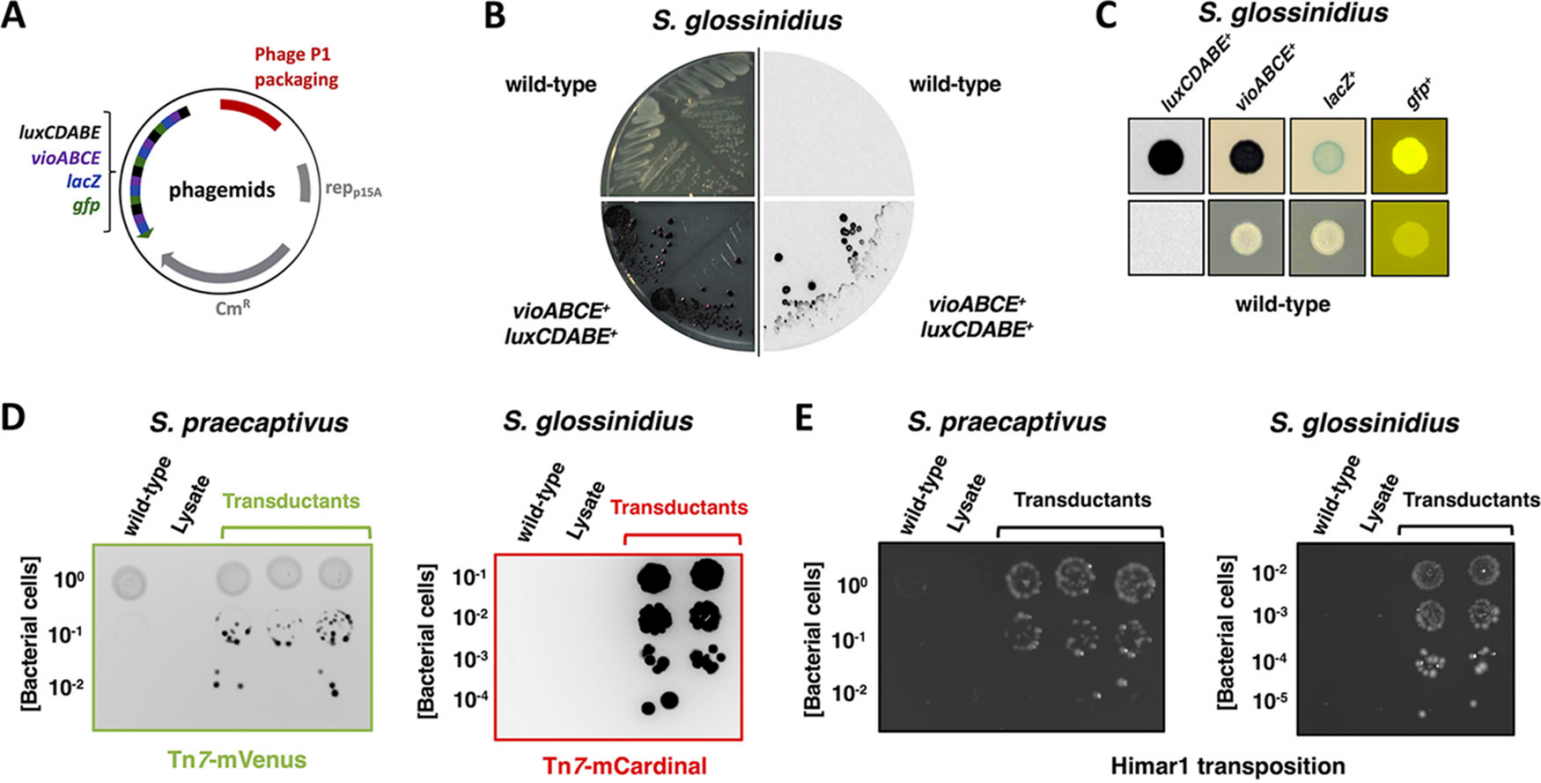
Transduction of P1 phagemids into *Sodalis* species. (A) Schematic representation of replication-competent P1 phagemids encoding a number of phenotypic markers (38). (B) Comparison of wild-type (top quadrants) and *S. glossinidius* transductants (lower quadrants) carrying P1 phagemid BBa_J72114-BBa_J72104 (38). Transductant colonies are purple due to the expression of violacein biosynthetic genes (lower left quadrant), and produce light due to the expression of bioluminescence genes (lower right quadrant). (C) Macrocolonies derived from wild-type *S. glossinidius* (bottom row) and transductants carrying P1 phagemids BBa_J72114-BBa_J72104 (*luxCDABE^+^* and *vioABCE^+^*), BBa_J72114-BBa_J72100 (*lacZ^+^*), or BBa_J72113-BBa_J72152 (*gfp^+^*) (top row). (D) Transduction of pP1-Tn*7*-mVenus into *S. praecaptivus* (left-hand side plate) and pP1-Tn*7*-mCardinal into *S. glossinidius* (right-hand side plate). (E) Transduction of phagemid encoding a Himar1 transposition system (pP1-Himar) into *S. praecaptivus* (left-hand side plate) and *S. glossinidius* (right-hand side plate).

The lack of genetic tools available for the manipulation of *Sodalis* species, specifically *S. glossinidius*, prompted us to explore P1 as a plasmid DNA delivery tool for these bacteria. As a proof of principle, we used the aforementioned general technique to transfer a number of P1 phagemids (38) (Fig. 5A) into *S. glossinidius*. We were able to recover transductants expressing an array of phenotypic traits encoded in the phagemids. These traits included light production (*luxCDABE* genes), violacein pigment synthesis (*vioABCE*), β-galactosidase activity (*lacZ*), or green fluorescence (*gfp*) (Fig. 5B and C). To expand the tool set available for the modification of *Sodalis* species, we constructed two phagemids for tagging bacterial chromosomes with fluorescent genes at the Tn*7* attachment site (40) and a suicide phagemid encoding a Himar1 transposition system for random mutagenesis (41) (Fig. S2). Following packaging into P1 virions in an E. coli P1CM*clr*-100(ts) lysogen, these phagemids were efficiently delivered to *S. glossinidius* and *S. praecaptivus* (Fig. 5D and E). Together, these results establish that bacteriophage P1 can be used to efficiently deliver replication-competent and suicide vectors into *S. glossinidius* and *S. praecaptivus* through a “guided transduction” strategy.

10.1128/mSphere.01331-20.2 FIG S2 Schematic representation of P1 phagemids used for tagging the bacterial chromosome at the Tn*7* attachment site(s) with genes encoding the fluorescent proteins mCardinal (pP1-Tn*7*-mCardinal) or mVenus (pP1-Tn*7*-mVenus) and the suicide P1 phagemid for random mutagenesis with a Himar1 transposition system (pP1-Himar). Download FIG S2, TIF file, 2.6 MB. Copyright © 2021 Keller et al. 2021 Keller et al. https://creativecommons.org/licenses/by/4.0/ This content is distributed under the terms of the Creative Commons Attribution 4.0 International license.

## DISCUSSION

In the current study, we demonstrate that bacteriophage P1 can infect, lysogenize, and promote transduction in two species of the genus *Sodalis*. We show that P1 can mediate generalized transduction in *S. praecaptivus* (Fig. 4), and we establish that this bacteriophage can be used for the delivery of plasmids and suicide vectors for the genetic manipulation of *S. glossinidius* and *S. praecaptivus* (Fig. 5). While these results constitute a significant advance in the development of genetic modification tools to study these bacterial species, they also clear the way for the implementation of P1-based DNA delivery systems to uncultured *Sodalis* species (42–48) and Gram-negative insect endosymbionts belonging to other genera.

Whereas *S. praecaptivus* can be genetically engineered with relative ease, the ability of P1 to mediate generalized transduction provides a number of applications for the manipulation of this bacterium. For instance, although *S. praecaptivus* can be readily modified by recombineering functions of phage λ (λ-Red) (49–51), the use of this technique has two major drawbacks. First, the expression of recombineering functions can be mutagenic (52). This can potentially produce confounding results in subsequent experiments as phenotypes associated with a particular engineered modification may actually result from secondary mutation(s). Second, typical temperature-sensitive plasmids (rep_pSC101_^ts^ *ori*) harboring recombineering functions cannot be cured from *S. praecaptivus* by propagating cells at nonpermissive temperatures (≥37°C) in the absence of plasmid selection (our unpublished results). The inability to cure these plasmids can increase the chances of secondary mutations through leaky expression of recombineering functions and hinder the use of plasmids from the same incompatibility group in downstream genetic analyses. Importantly, both of these issues can be overcome by P1-mediated generalized transduction. That is, genomic DNA fragments engineered using λ-Red can be transferred to naive *S. praecaptivus* cells that lack recombineering plasmids and, therefore, have not been exposed to potential mutagenic events (Fig. 4B).

In contrast, the establishment of P1-mediated transduction provides a considerable advancement in our ability to genetically manipulate *S. glossinidius*. This is because *S. glossinidius* is recalcitrant to DNA transformation by standard techniques such as heat shock and electroporation (29, 30, 53). Whereas we have recently developed a method for DNA transfer to *S. glossinidius* via conjugation (30), P1-mediated transduction provides an alternative, simpler method for the introduction of exogenous DNA into this bacterium. Altered chromosomal fragments, replication-competent plasmids carrying an array of functions, and suicide vectors engineered for allelic replacement or containing transposition systems can be quickly transduced into *S. glossinidius* in a simple protocol. Given the large DNA packaging capability of P1 (up to 100 kbp) (5), this bacteriophage can be efficiently used for a variety of applications, including the delivery of bacterial artificial chromosomes (bacterial artificial chromosome [BAC] vectors) or large plasmids encoding multiple genome editing CRISPR systems (54) that are not easily transferred by conjugation (30). Additionally, the P1 packing sequence can be incorporated into DNA fragments used in insertional mutagenesis (39), enabling rapid and efficient combination of mutations via P1 “guided transduction.” This approach can greatly facilitate the implementation of several analyses (e.g., complementation and epistasis) to identify and dissect genetic components and pathways governing bacterial behaviors and interactions with eukaryotic hosts.

Beyond the genus *Sodalis*, the results highlighted in this study have potential broad implications for the genetic modification of uncultured Gram-negative insect endosymbionts. In Gram-negative bacteria, the LPS is the major structural constituent of the outer leaflet of the outer membrane. The LPS is composed of a highly conserved lipid A “anchor,” a conserved core polysaccharide and, sometimes, a hypervariable outer component designated O antigen (see Fig. S1A in the supplemental material) (76). Bacteriophage P1 has a broad host range, in part, because it recognizes, as its host receptor, structural features of the conserved LPS core (8). In addition to E. coli and several species of the *Gammaproteobacteria*, P1 has been shown to be capable of infecting various members within the *Alpha*, *Beta*-, and Deltaproteobacteria, and even bacterial species residing outside the *Proteobacteria* phylum such as *Flavobacterium* sp. strain M64 (8–12).

Notably, a large number of economically important insect species—including several disease vectors of animals and plants—harbor maternally inherited, Gram-negative bacterial endosymbionts (24, 25). However, unlike *S. glossinidius* and a handful of other species, the vast majority of these endosymbionts have not been isolated in pure culture (14, 19, 55–59). This is because these bacteria undergo a process of genome degeneration and size reduction during the course of long-term evolution and specialization within their eukaryotic insect hosts. This process leads to the loss of many physiological functions that are required for replication outside the host (24, 25, 27, 29). Notably, classical protocols of bacterial genetics require the manipulation of large numbers of cells and the subsequent isolation of rare genetic events as bacterial colonies on selective agar plates. Consequently, the implementation of genetics for the study of insect endosymbionts has remained scarce and limited to species that can grow in axenic culture, form colonies on agar plates, and are receptive to exogenous DNA (29, 30).

The ability of bacteriophage P1 to deliver DNA to a large number of bacterial species suggests a clear method in which the requirement for culturing may be bypassed. That is, similar to viral vectors that are commonly utilized in gene therapy in mammalians (60), P1 could be used to deliver DNA to bacterial endosymbionts inside their insect hosts. Specifically, insects could be microinjected (23, 27, 61–63) with P1 virions packaged with recombinant DNA. The establishment of successful P1 infections and subsequent enrichment of transductant endsymbionts to near-homogeneous or clonal populations could be attained by making use of phenotypic markers (Fig. 5) and implementing antibiotic selection regiments in insects (64–69). Whereas this approach would preferentially target recently acquired endosymbionts by virtue of their ability to exist intracellularly and extracellularly in various insect tissues (21–23, 25), ancient obligate intracellular endosymbionts could also be subjected to infection and genetic modification via P1, if they transiently exit host cells (70).

The results presented in this study pave the way for the development of tractable genetic systems for *S. glossinidius* and, potentially, a myriad of Gram-negative bacterial endosymbionts of insects. While this may empower the use of genetics to study these obscure bacteria, it also has clear translational applications. P1-mediated DNA delivery into insect endosymbionts may allow the engineering of bacterial traits aimed at modifying aspects of insect ecology (29, 30), mitigating their burden on economic activities and human health.

## MATERIALS AND METHODS

### Microbial strains, phages, plasmids, and growth conditions.

Microbial strains, phages, and plasmids used in this study are presented in Table S1 in the supplemental material. Unless indicated, all Escherichia coli strains were propagated at 30, 37, or 42°C in Luria-Bertani (LB) broth or agar (1.5% [wt/vol]). Sodalis glossinidius was grown at 27°C in brain heart infusion broth supplemented with 10 mM MgCl_2_ (BHI) or on brain heart infusion agar (1.2% [wt/vol]) supplemented with 10 mM MgCl_2_ (BHI agar). S. glossinidius was also propagated on BHI agar plates supplemented with 10% defibrinated horse blood (BHIB). Sodalis praecaptivus was grown at 30, 39, or 42°C in Luria-Bertani broth or agar (1.5% [wt/vol]) lacking sodium chloride. For experiments involving P1 infection or generation of lysates, the growth medium was supplemented with CaCl_2_ and MgCl_2_ to a final concentration of 10 mM, respectively. Growth of S. glossinidius on BHIB agar plates was carried out under microaerophilic conditions, which was achieved either using BD GasPak EZ Campy Gas Generating sachets or a gas mixture (5% oxygen and 95% CO_2_). For all strains, growth in liquid medium was carried out in shaking water bath incubators with aeration (250 rpm). When required, medium was supplemented with ampicillin (100 μg/ml), chloramphenicol (20 μg/ml for E. coli or *S. praecaptivus* and 10 μg/ml for *S. glossinidius*), kanamycin (50 μg/ml for E. coli and 25 μg/ml for *S. glossinidius* or *S. praecaptivus*). Arabinose was used at a concentration of 0.5 or 1% (wt/vol); 5-bromo-4-chloro-3-indolyl-β-d-galactopyranoside (X-Gal) was used at a concentration of 100 μg/ml.

10.1128/mSphere.01331-20.3 TABLE S1 Microbial strains, phages, and plasmids used in this study. Download Table S1, XLSX file, 0.01 MB. Copyright © 2021 Keller et al. 2021 Keller et al. https://creativecommons.org/licenses/by/4.0/ This content is distributed under the terms of the Creative Commons Attribution 4.0 International license.

### Lipopolysaccharide extraction and detection.

Extraction of lipopolysaccharide (LPS) from *S. glossinidius* and *S. praecaptivus* cultures was carried out as described previously (71). Extracted samples were separated in a NuPAGE 10% Bis-Tris gel in NuPAGE MES SDS Running (ThermoFisher Scientific). LPS in gels were stained with ProteoSilver silver stain kit (Sigma-Aldrich).

### Construction of phagemid pP1-Tn*7-*mCardinal.

Oligonucleotide sequences used in this study are presented in Table S2. Phusion high-fidelity DNA polymerase (New England BioLabs) was used in PCRs with primers 469 and 470 and plasmid BBa_J72113-BBa_J72152 (38) as the template. The PCR product was ligated into pMRE-Tn*7*-163 (72), previously digested with SbfI, using NEBuilder HiFi DNA Assembly (New England BioLabs). The integrity of the construct was verified by DNA sequencing and the ability to be efficiently transduced by P1 particles.

10.1128/mSphere.01331-20.4 TABLE S2 Oligonucleotide sequences used in this study. Download Table S2, XLSX file, 0.01 MB. Copyright © 2021 Keller et al. 2021 Keller et al. https://creativecommons.org/licenses/by/4.0/ This content is distributed under the terms of the Creative Commons Attribution 4.0 International license.

### Construction of phagemid pP1-Tn*7*-mVenus.

Oligonucleotide sequences used in this study are presented in Table S2. Phusion high-fidelity DNA polymerase (New England BioLabs) was used in PCRs with primers 469 and 470 and plasmid BBa_J72113-BBa_J72152 (38) as the template. The PCR product was ligated into pMRE-Tn*7*-166 (72), previously digested with SbfI, using NEBuilder HiFi DNA Assembly (New England BioLabs). The integrity of the construct was verified by DNA sequencing and the ability to be efficiently transduced by P1 particles.

### Construction of phagemid pP1-Himar.

Oligonucleotide sequences used in this study are presented in Table S2. Phusion high-fidelity DNA polymerase (New England BioLabs) was used in PCRs with primers 475 and 476 and plasmid BBa_J72113-BBa_J72152 (38) as the template. The PCR product was ligated into pMarC9-R6k (73), previously digested with EcoRI and HindIII, using NEBuilder HiFi DNA Assembly (New England BioLabs). The integrity of the construct was verified by DNA sequencing and the ability to be efficiently transduced by P1 particles.

### Recombineering procedure for S. praecaptivus.

Oligonucleotide sequences used in this study are presented in Table S2. An *S. praecaptivus* strain harboring plasmid pSIM6 (35) was grown overnight in LB broth supplemented with 100 μg/ml of ampicillin at 30°C and 250 rpm. Cells were diluted (1:100) in 30 ml of the same medium and grown to an optical density at 600 nm (OD_600_) between 0.45 and 0.5. The culture flask was then grown in a water bath at 42°C and 250 rpm for 25 min. Cells were immediately transferred to a 50-ml conical tube, collected by centrifugation (7,000 rpm for 2.5 min at 4°C), and resuspended in 40 ml of ice-cold deionized H_2_O (dH_2_O). Cells were collected again by centrifugation, and this washing procedure was repeated a second time. Finally, cells were resuspended in 150 μl of ice-cold dH_2_O. Homologous recombination was obtained by electroporating 70 μl of cell suspension with 10 μl of purified PCR products generated with primers 251 and 252 (*galU*::*Kn*) or primers 84 and 85 (*rpoS-HA*::*Cm*) and plasmids pKD4 and pKD3 (74) as the templates, respectively.

### Preparation of phage lysates derived from EMG16 P1 lysogens.

Lysates from E. coli EMG16 harboring selected phagemids were prepared following arabinose induction as described previously (38).

### Preparation of stocks of P1*vir* phage lysates.

Stocks of P1*vir* phage lysates were prepared by infecting E. coli MG1655 (75), as described previously (5).

### Preparation of phage lysates derived from P1CM*clr*-100(ts) lysogens.

E. coli P1CM*clr*-100(ts) lysogens were grown overnight at 30°C. Cultures were diluted (1:100 [vol/vol]) into fresh medium and grown to an OD_600_ value of 0.3 to 0.4. Subsequently, cultures were shifted to 42°C and propagated until extensive cell lysis (3 to 4 h). At times, cultures were allowed to grow at 42°C for 16 h prior to the preparation of lysates. Partially lysed cells were disrupted by vortexing the cultures following the addition of chloroform (1 volume of chloroform per 100 volumes of culture). Cell debris was removed by centrifugation (12 min, 4,000 rpm, room temperature), and the supernatant was passed through a 0.22-μm polyethersulfone membrane filter. S. praecaptivus P1CM*clr*-100(ts) lysogens were prepared as described above, except that cells were grown for 2 h at 42°C and 16 h at 37°C prior to lysate preparation. S. glossinidius P1CM*clr*-100(ts) lysogens were grown in BHI to an OD_600_ of 0.4. Cultures were heat shocked at 37°C for 2 h. The cultures were treated with chloroform (1 volume of chloroform per 100 volumes of culture) and processed as described for E. coli and *S. praecaptivus*.

### Infection by P1*vir*, P1CM*clr*-100(ts), and P1 transducing particles.

Following overnight growth in LB, E. coli cultures were diluted in fresh LB supplemented with 10 mM CaCl_2_ and MgCl_2_ to an OD_600_ of 1. One-milliliter aliquots of these cell solutions were incubated for 30 min at 30°C in the presence or absence of various concentrations of P1 lysate. Cells were subsequently collected by centrifugation (1 min, 13,000 rpm, room temperature), and the supernatants were replaced by 1 ml of LB containing 5 mM sodium citrate. Cells were grown for 1 h at 30°C and 250 rpm prior to plating. S. praecaptivus cells grown overnight in LB were collected by centrifugation (1 min, 13,000 rpm, room temperature) and resuspended in fresh LB supplemented with 10 mM CaCl_2_ and MgCl_2_. One milliliter of these resuspended solutions was incubated for 30 min at 30°C in the presence or absence of various concentrations of P1 lysate. Cells were collected by centrifugation (1 min, 13,000 rpm, room temperature), and the supernatants were replaced by 1 ml of LB containing 5 mM sodium citrate. Cells were plated following 1 h of growth at 30°C and 250 rpm. S. glossinidius cells were grown in BHI for 3 to 5 days to an OD_600_ of ≈0.5. Cells were collected by centrifugation and concentrated to an OD_600_ of 1. One milliliter of concentrated cultures was incubated for 60 min at 30°C in the presence or absence of various concentrations of P1 lysate. Cells were collected by centrifugation (1 min, 13,000 rpm, room temperature), and the supernatants were replaced by 10 ml of BHI. Cultures were incubated overnight at 27°C overnight with shaking prior to plating.

### Curing of pP1-Tn*7* phagemids.

Transduction of pP1-Tn*7* phagemids into *S. glossinidius* and *S. praecaptivus* was initially selected on plates containing ampicillin (Fig. 5D; see also Fig. S2 in the supplemental material). Because episomes harboring rep_pSC101_^ts^ origins of replication are not easily cured from *S. praecaptivus* (see Discussion), the curing of phagemids was performed only in *S. glossinidius*. The strategy used to identify *S. glossinidius* clones lacking phagemids was similar to the one adopted elsewhere (53). Briefly, to identify *S. glossinidius* clones that contained the chloramphenicol-resistant marker at the Tn*7* attachment site and had lost the ampicillin-resistant plasmid, cultures were propagated in BHI containing chloramphenicol and 1% arabinose. After four passages, cells were diluted and plated. Single colonies were screened for sensitivity to ampicillin. Transposon insertion at the Tn*7* attachment site was verified by PCR with primers 1018 and 1019.

### Image acquisition, analysis, and manipulation.

DNA agarose gel electrophoresis and bacterial colonies, with the exception of *S. glossinidius* macrocolonies, were detected using an Amersham Imager 680 (GE Healthcare). *S. glossinidius* macrocolonies expressing green fluorescent protein (GFP) were detected using a dark reader (Clare Chemical Research) and documented with an iPhone. When oversaturated, the intensity of signals in images were adjusted across the entire images using Preview (Apple).

## References

[1] Hendrix RW, Smith MC, Burns RN, Ford ME, Hatfull GF. 1999. Evolutionary relationships among diverse bacteriophages and prophages: all the world's a phage. Proc Natl Acad Sci U S A 96:2192–2197. doi:10.1073/pnas.96.5.2192. 10051617 PMC26759

[2] Touchon M, Moura de Sousa JA, Rocha EP. 2017. Embracing the enemy: the diversification of microbial gene repertoires by phage-mediated horizontal gene transfer. Curr Opin Microbiol 38:66–73. doi:10.1016/j.mib.2017.04.010. 28527384

[3] Soucy SM, Huang J, Gogarten JP. 2015. Horizontal gene transfer: building the web of life. Nat Rev Genet 16:472–482. doi:10.1038/nrg3962. 26184597

[4] Hershey AD, Chase M. 1952. Independent functions of viral protein and nucleic acid in growth of bacteriophage. J Gen Physiol 36:39–56. doi:10.1085/jgp.36.1.39. 12981234 PMC2147348

[5] Thomason LC, Costantino N, Court DL. 2007. *E. coli* genome manipulation by P1 transduction. Curr Protoc Mol Biol Chapter 1:Unit 1.17. doi:10.1002/0471142727.mb0117s79. 18265391

[6] Bertani G. 1951. Studies on lysogenesis. I. The mode of phage liberation by lysogenic *Escherichia coli*. J Bacteriol 62:293–300. doi:10.1128/JB.62.3.293-300.1951. 14888646 PMC386127

[7] Lennox ES. 1955. Transduction of linked genetic characters of the host by bacteriophage P1. Virology 1:190–206. doi:10.1016/0042-6822(55)90016-7. 13267987

[8] Yarmolinsky MB, Sternberg N. 1988. Bacteriophage P1, p 291–438. In Calendar R (ed), The bacteriophages, vol 1. Plenum Press, New York, NY.

[9] Goldberg RB, Bender RA, Streicher SL. 1974. Direct selection for P1-sensitive mutants of enteric bacteria. J Bacteriol 118:810–814. doi:10.1128/JB.118.3.810-814.1974. 4598005 PMC246826

[10] Ornellas EP, Stocker BA. 1974. Relation of lipopolysaccharide character to P1 sensitivity in *Salmonella typhimurium*. Virology 60:491–502. doi:10.1016/0042-6822(74)90343-2. 4602344

[11] Kaiser D, Dworkin M. 1975. Gene transfer to myxobacterium by *Escherichia coli* phage P1. Science 187:653–654. doi:10.1126/science.803710. 803710

[12] Murooka Y, Harada T. 1979. Expansion of the host range of coliphage P1 and gene transfer from enteric bacteria to other Gram-negative bacteria. Appl Environ Microbiol 38:754–757. doi:10.1128/AEM.38.4.754-757.1979. 395900 PMC243574

[13] Streicher S, Gurney E, Valentine RC. 1971. Transduction of the nitrogen-fixation genes in *Klebsiella pneumoniae*. Proc Natl Acad Sci U S A 68:1174–1177. doi:10.1073/pnas.68.6.1174. 5288365 PMC389145

[14] O'Connor KA, Zusman DR. 1983. Coliphage P1-mediated transduction of cloned DNA from *Escherichia coli* to *Myxococcus xanthus*: use for complementation and recombinational analyses. J Bacteriol 155:317–329. doi:10.1128/JB.155.1.317-329.1983. 6305916 PMC217683

[15] Downard JS. 1988. Tn5-mediated transposition of plasmid DNA after transduction to *Myxococcus xanthus*. J Bacteriol 170:4939–4941. doi:10.1128/jb.170.10.4939-4941.1988. 2844730 PMC211541

[16] Wolf-Watz H, Portnoy DA, Bölin I, Falkow S. 1985. Transfer of the virulence plasmid of *Yersinia pestis* to *Yersinia pseudotuberculosis*. Infect Immun 48:241–253. doi:10.1128/IAI.48.1.241-243.1985. 3884511 PMC261941

[17] Westwater C, Schofield DA, Schmidt MG, Norris JS, Dolan JW. 2002. Development of a P1 phagemid system for the delivery of DNA into Gram-negative bacteria. Microbiology (Reading) 148:943–950. doi:10.1099/00221287-148-4-943. 11932441

[18] Butela K, Lawrence JG. 2012. Genetic manipulation of pathogenicity loci in non-Typhimurium *Salmonella*. J Microbiol Methods 91:477–482. doi:10.1016/j.mimet.2012.09.013. 23041268 PMC3658464

[19] Dale C, Maudlin I. 1999. *Sodalis* gen. nov. and *Sodalis glossinidius* sp. nov., a microaerophilic secondary endosymbiont of the tsetse fly *Glossina morsitans morsitans*. Int J Syst Bacteriol 49:267–275. doi:10.1099/00207713-49-1-267. 10028272

[20] Chari A, Oakeson KF, Enomoto S, Jackson DG, Fisher MA, Dale C. 2015. Phenotypic characterization of *Sodalis praecaptivus* sp. nov., a close non-insect-associated member of the *Sodalis*-allied lineage of insect endosymbionts. Int J Syst Evol Microbiol 65:1400–1405. doi:10.1099/ijs.0.000091. 25782768 PMC4635462

[21] Aksoy S, Chen X, Hypsa V. 1997. Phylogeny and potential transmission routes of midgut-associated endosymbionts of tsetse (Diptera: Glossinidae). Insect Mol Biol 6:183–190. doi:10.1111/j.1365-2583.1997.tb00086.x. 9099582

[22] Cheng Q, Aksoy S. 1999. Tissue tropism, transmission and expression of foreign genes *in vivo* in midgut symbionts of tsetse flies. Insect Mol Biol 8:125–132. doi:10.1046/j.1365-2583.1999.810125.x. 9927181

[23] De Vooght L, Caljon G, Van Hees J, Van Den Abbeele J. 2015. Paternal transmission of a secondary symbiont during mating in the viviparous tsetse fly. Mol Biol Evol 32:1977–1980. doi:10.1093/molbev/msv077. 25851957 PMC4833065

[24] McCutcheon JP, Boyd BM, Dale C. 2019. The life of an insect endosymbiont from the cradle to the grave. Curr Biol 29:R485–R495. doi:10.1016/j.cub.2019.03.032. 31163163

[25] Moran NA, McCutcheon JP, Nakabachi A. 2008. Genomics and evolution of heritable bacterial symbionts. Annu Rev Genet 42:165–190. doi:10.1146/annurev.genet.41.110306.130119. 18983256

[26] Toh H, Weiss BL, Perkin SA, Yamashita A, Oshima K, Hattori M, Aksoy S. 2006. Massive genome erosion and functional adaptations provide insights into the symbiotic lifestyle of *Sodalis glossinidius* in the tsetse host. Genome Res 16:149–156. doi:10.1101/gr.4106106. 16365377 PMC1361709

[27] Pontes MH, Smith KL, De Vooght L, Van Den Abbeele J, Dale C. 2011. Attenuation of the sensing capabilities of PhoQ in transition to obligate insect-bacterial association. PLoS Genet 7:e1002349. doi:10.1371/journal.pgen.1002349. 22072980 PMC3207850

[28] Clayton AL, Oakeson KF, Gutin M, Pontes A, Dunn DM, von Niederhausern AC, Weiss RB, Fisher M, Dale C. 2012. A novel human-infection-derived bacterium provides insights into the evolutionary origins of mutualistic insect-bacterial symbioses. PLoS Genet 8:e1002990. doi:10.1371/journal.pgen.1002990. 23166503 PMC3499248

[29] Pontes MH, Dale C. 2006. Culture and manipulation of insect facultative symbionts. Trends Microbiol 14:406–412. doi:10.1016/j.tim.2006.07.004. 16875825

[30] Kendra CG, Keller CM, Bruna RE, Pontes MH. 2020. Conjugal DNA transfer in the maternally inherited symbiont of tsetse flies *Sodalis glossinidius*. mSphere 5:e00864-20. doi:10.1128/mSphere.00864-20. 33148821 PMC7643829

[31] Rosner JL. 1972. Formation, induction, and curing of bacteriophage P1 lysogens. Virology 48:679–689. doi:10.1016/0042-6822(72)90152-3. 4555608

[32] Wilkinson RG, Stocker BA. 1968. Genetics and cultural properties of mutants of *Salmonella typhimurium* lacking glucosyl or galactosyl lipopolysaccharide transferases. Nature 217:955–957. doi:10.1038/217955a0. 4868316

[33] Osborn MJ. 1968. Biochemical characterization of mutants of *Salmonella typhimurium* lacking glucosyl or galactosyl lipopolysaccharide transferases. Nature 217:957–960. doi:10.1038/217957a0. 4868317

[34] Ikeda H, Tomizawa JI. 1965. Transducing fragments in generalized transduction by phage P1. I. Molecular origin of the fragments. J Mol Biol 14:85–109. doi:10.1016/s0022-2836(65)80232-7. 5883923

[35] Datta S, Costantino N, Court DL. 2006. A set of recombineering plasmids for gram-negative bacteria. Gene 379:109–115. doi:10.1016/j.gene.2006.04.018. 16750601

[36] Sternberg N, Coulby J. 1987. Recognition and cleavage of the bacteriophage P1 packaging site (*pac*). I. Differential processing of the cleaved ends *in vivo*. J Mol Biol 194:453–468. doi:10.1016/0022-2836(87)90674-7. 3305962

[37] Sternberg N, Coulby J. 1987. Recognition and cleavage of the bacteriophage P1 packaging site (*pac*). II. Functional limits of *pac* and location of *pac* cleavage termini. J Mol Biol 194:469–479. doi:10.1016/0022-2836(87)90675-9. 3625770

[38] Kittleson JT, DeLoache W, Cheng HY, Anderson JC. 2012. Scalable plasmid transfer using engineered P1-based phagemids. ACS Synth Biol 1:583–589. doi:10.1021/sb300054p. 23656280 PMC3804010

[39] Huang H, Masters M. 2014. Bacteriophage P1 *pac* sites inserted into the chromosome greatly increase packaging and transduction of *Escherichia coli* genomic DNA. Virology 468-470:274–282. doi:10.1016/j.virol.2014.07.029. 25213407

[40] Peters JE, Craig NL. 2001. Tn*7*: smarter than we thought. Nat Rev Mol Cell Biol 2:806–814. doi:10.1038/35099006. 11715047

[41] Rubin EJ, Akerley BJ, Novik VN, Lampe DJ, Husson RN, Mekalanos JJ. 1999. *In vivo* transposition of mariner-based elements in enteric bacteria and mycobacteria. Proc Natl Acad Sci U S A 96:1645–1650. doi:10.1073/pnas.96.4.1645. 9990078 PMC15546

[42] Heddi A, Charles H, Khatchadourian C, Bonnot G, Nardon P. 1998. Molecular characterization of the principal symbiotic bacteria of the weevil *Sitophilus oryzae*: a peculiar G+C content of an endocytobiotic DNA. J Mol Evol 47:52–61. doi:10.1007/pl00006362. 9664696

[43] Fukatsu T, Koga R, Smith WA, Tanaka K, Nikoh N, Sasaki-Fukatsu K, Yoshizawa K, Dale C, Clayton DH. 2007. Bacterial endosymbiont of the slender pigeon louse, *Columbicola columbae*, allied to endosymbionts of grain weevils and tsetse flies. Appl Environ Microbiol 73:6660–6668. doi:10.1128/AEM.01131-07. 17766458 PMC2075037

[44] Nováková E, Hypsa V. 2007. A new *Sodalis* lineage from bloodsucking fly *Craterina melbae* (Diptera, Hippoboscoidea) originated independently of the tsetse flies symbiont *Sodalis glossinidius*. FEMS Microbiol Lett 269:131–135. doi:10.1111/j.1574-6968.2006.00620.x. 17227456

[45] Chrudimský T, Husník F, Nováková E, Hypša V. 2012. *Candidatus* Sodalis melophagi sp. nov.: phylogenetically independent comparative model to the tsetse fly symbiont *Sodalis glossinidius*. PLoS One 7:e40354. doi:10.1371/journal.pone.0040354. 22815743 PMC3398932

[46] Smith WA, Oakeson KF, Johnson KP, Reed DL, Carter T, Smith KL, Koga R, Fukatsu T, Clayton DH, Dale C. 2013. Phylogenetic analysis of symbionts in feather-feeding lice of the genus *Columbicola*: evidence for repeated symbiont replacements. BMC Evol Biol 13:109. doi:10.1186/1471-2148-13-109. 23725492 PMC3724504

[47] Santos-Garcia D, Silva FJ, Morin S, Dettner K, Kuechler SM. 2017. The all-rounder *Sodalis*: a new bacteriome-associated endosymbiont of the lygaeoid bug *Henestaris halophilus* (Heteroptera: Henestarinae) and a critical examination of its evolution. Genome Biol Evol 9:2893–2910. doi:10.1093/gbe/evx202. 29036401 PMC5737371

[48] Šochová E, Husník F, Nováková E, Halajian A, Hypša V. 2017. *Arsenophonus* and *Sodalis* replacements shape evolution of symbiosis in louse flies. PeerJ 5:e4099. doi:10.7717/peerj.4099. 29250466 PMC5729840

[49] Clayton AL, Enomoto S, Su Y, Dale C. 2017. The regulation of antimicrobial peptide resistance in the transition to insect symbiosis. Mol Microbiol 103:958–972. doi:10.1111/mmi.13598. 27987256 PMC5344769

[50] Enomoto S, Chari A, Clayton AL, Dale C. 2017. Quorum sensing attenuates virulence in *Sodalis praecaptivus*. Cell Host Microbe 21:629–636.e5. doi:10.1016/j.chom.2017.04.003. 28494244 PMC5542680

[51] Thomason LC, Sawitzke JA, Li X, Costantino N, Court DL. 2014. Recombineering: genetic engineering in bacteria using homologous recombination. Curr Protoc Mol Biol 106:1.16.1–1.16.39. doi:10.1002/0471142727.mb0116s106. 24733238

[52] Murphy KC, Campellone KG. 2003. Lambda Red-mediated recombinogenic engineering of enterohemorrhagic and enteropathogenic *E. coli*. BMC Mol Biol 4:11. doi:10.1186/1471-2199-4-11. 14672541 PMC317293

[53] Pontes MH, Dale C. 2011. Lambda red-mediated genetic modification of the insect endosymbiont *Sodalis glossinidius*. Appl Environ Microbiol 77:1918–1920. doi:10.1128/AEM.02166-10. 21216910 PMC3067283

[54] Adiego-Pérez B, Randazzo P, Daran JM, Verwaal R, Roubos JA, Daran-Lapujade P, van der Oost J. 2019. Multiplex genome editing of microorganisms using CRISPR-Cas. FEMS Microbiol Lett 366:fnz086. doi:10.1093/femsle/fnz086. 31087001 PMC6522427

[55] Hypsa V, Dale C. 1997. *In vitro* culture and phylogenetic analysis of "*Candidatus* Arsenophonus triatominarum," an intracellular bacterium from the triatomine bug, *Triatoma infestans*. Int J Syst Bacteriol 47:1140–1144. doi:10.1099/00207713-47-4-1140. 9336921

[56] Sabri A, Leroy P, Haubruge E, Hance T, Frère I, Destain J, Thonart P. 2011. Isolation, pure culture and characterization of *Serratia symbiotica* sp. nov., the R-type of secondary endosymbiont of the black bean aphid *Aphis fabae*. Int J Syst Evol Microbiol 61:2081–2088. doi:10.1099/ijs.0.024133-0. 20870890

[57] Dale C, Beeton M, Harbison C, Jones T, Pontes M. 2006. Isolation, pure culture, and characterization of "*Candidatus* Arsenophonus arthropodicus," an intracellular secondary endosymbiont from the hippoboscid louse fly *Pseudolynchia canariensis*. Appl Environ Microbiol 72:2997–3004. doi:10.1128/AEM.72.4.2997-3004.2006. 16598007 PMC1449044

[58] Brandt JW, Chevignon G, Oliver KM, Strand MR. 2017. Culture of an aphid heritable symbiont demonstrates its direct role in defence against parasitoids. Proc Biol Soc 284:20171925. doi:10.1098/rspb.2017.1925. PMC5698653 29093227

[59] Masson F, Calderon Copete S, Schüpfer F, Garcia-Arraez G, Lemaitre B. 2018. *In vitro* culture of the insect endosymbiont *Spiroplasma poulsonii* highlights bacterial genes Iinvolved in host-symbiont interaction. mBio 9:e00024-18. doi:10.1128/mBio.00024-18. 29559567 PMC5874924

[60] Kay MA, Glorioso JC, Naldini L. 2001. Viral vectors for gene therapy: the art of turning infectious agents into vehicles of therapeutics. Nat Med 7:33–40. doi:10.1038/83324. 11135613

[61] Boyle L, O'Neill SL, Robertson HM, Karr TL. 1993. Interspecific and intraspecific horizontal transfer of *Wolbachia* in *Drosophila*. Science 260:1796–1799. doi:10.1126/science.8511587. 8511587

[62] Oliver KM, Russell JA, Moran NA, Hunter MS. 2003. Facultative bacterial symbionts in aphids confer resistance to parasitic wasps. Proc Natl Acad Sci U S A 100:1803–1807. doi:10.1073/pnas.0335320100. 12563031 PMC149914

[63] Doremus MR, Smith AH, Kim KL, Holder AJ, Russell JA, Oliver KM. 2018. Breakdown of a defensive symbiosis, but not endogenous defences, at elevated temperatures. Mol Ecol 27:2138–2151. doi:10.1111/mec.14399. 29087015

[64] Wilkinson TL. 1998. The elimination of intracellular microorganisms from insects: an analysis of antibiotic-treatment in the pea aphid (*Acyrthosiphon pisum*). Comp Biochem Physiol Ser 119:871–881. doi:10.1016/S1095-6433(98)00013-0.

[65] Dale C, Welburn SC. 2001. The endosymbionts of tsetse flies: manipulating host-parasite interactions. Int J Parasitol 31:628–631. doi:10.1016/s0020-7519(01)00151-5. 11334953

[66] Dobson SL, Rattanadechakul W. 2001. A novel technique for removing *Wolbachia* infections from *Aedes albopictus* (Diptera: Culicidae). J Med Entomol 38:844–849. doi:10.1603/0022-2585-38.6.844. 11761383

[67] Koga R, Tsuchida T, Sakurai M, Fukatsu T. 2007. Selective elimination of aphid endosymbionts: effects of antibiotic dose and host genotype, and fitness consequences. FEMS Microbiol Ecol 60:229–239. doi:10.1111/j.1574-6941.2007.00284.x. 17313660

[68] Pais R, Lohs C, Wu Y, Wang J, Aksoy S. 2008. The obligate mutualist *Wigglesworthia glossinidia* influences reproduction, digestion, and immunity processes of its host, the tsetse fly. Appl Environ Microbiol 74:5965–5974. doi:10.1128/AEM.00741-08. 18689507 PMC2565960

[69] McLean AH, van Asch M, Ferrari J, Godfray HC. 2011. Effects of bacterial secondary symbionts on host plant use in pea aphids. Proc Biol Sci 278:760–766. doi:10.1098/rspb.2010.1654. 20843842 PMC3030854

[70] Zaidman-Rémy A, Vigneron A, Weiss BL, Heddi A. 2018. What can a weevil teach a fly, and reciprocally? Interaction of host immune systems with endosymbionts in *Glossina* and *Sitophilus*. BMC Microbiol 18:150. doi:10.1186/s12866-018-1278-5. 30470176 PMC6251153

[71] Davis MR, Jr, Goldberg JB. 2012. Purification and visualization of lipopolysaccharide from Gram-negative bacteria by hot aqueous-phenol extraction. J Vis Exp 2012:3916. doi:10.3791/3916. PMC3466933 22688346

[72] Schlechter RO, Jun H, Bernach M, Oso S, Boyd E, Muñoz-Lintz DA, Dobson RCJ, Remus DM, Remus-Emsermann MNP. 2018. Chromatic bacteria − a broad host-range plasmid and chromosomal insertion toolbox for fluorescent protein expression in bacteria. Front Microbiol 9:3052. doi:10.3389/fmicb.2018.03052. 30631309 PMC6315172

[73] Lee HH, Ostrov N, Wong BG, Gold MA, Khalil AS, Church GM. 2019. Functional genomics of the rapidly replicating bacterium *Vibrio natriegens* by CRISPRi. Nat Microbiol 4:1105–1113. doi:10.1038/s41564-019-0423-8. 30962569

[74] Datsenko KA, Wanner BL. 2000. One-step inactivation of chromosomal genes in *Escherichia coli* K-12 using PCR products. Proc Natl Acad Sci U S A 97:6640–6645. doi:10.1073/pnas.120163297. 10829079 PMC18686

[75] Blattner FR, Plunkett G, III, Bloch CA, Perna NT, Burland V, Riley M, Collado-Vides J, Glasner JD, Rode CK, Mayhew GF, Gregor J, Davis NW, Kirkpatrick HA, Goeden MA, Rose DJ, Mau B, Shao Y. 1997. The complete genome sequence of *Escherichia coli* K-12. Science 277:1453–1462. doi:10.1126/science.277.5331.1453. 9278503

[76] Silipo A, Molinaro A. 2010. The diversity of the core oligosaccharide in lipopolysaccharides. Subcell Biochem 53:69–99. doi:10.1007/978-90-481-9078-2_4. 20593263

