# Supplementary figures and images for: Genetic Modification of Sodalis Species by DNA Transduction
Source: mSphere. 2021 Feb 17;6(1):e01331-20. doi: 10.1128/mSphere.01331-20 (PMC8544901; doi:10.1128/mSphere.01331-20)

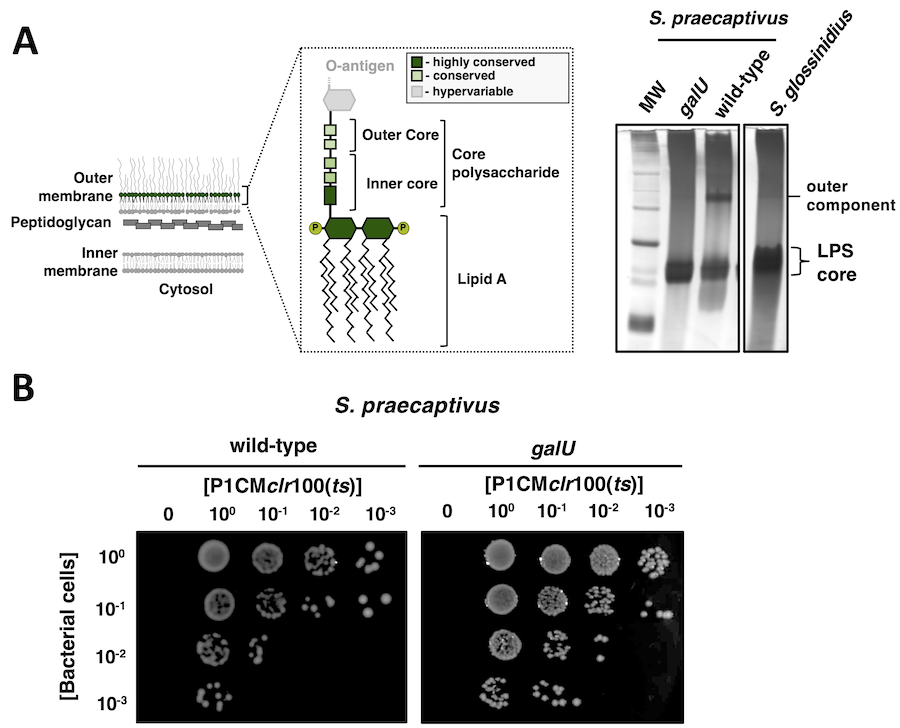

Supplement: FIG S1 [file msphere.01331-20-sf001.tif]

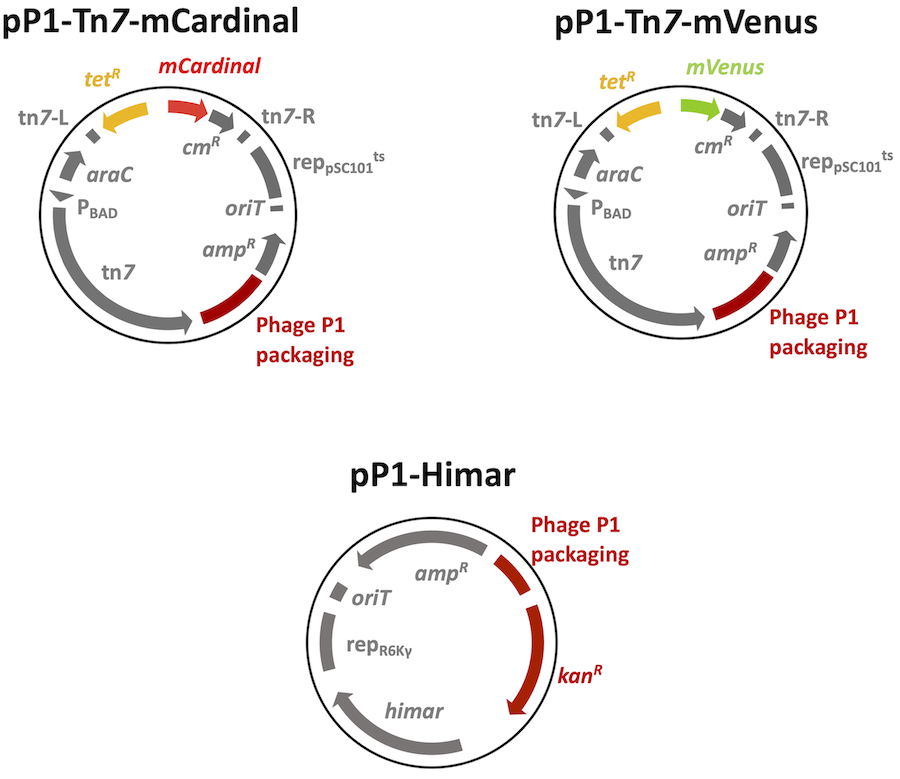

Supplement: FIG S2 [file msphere.01331-20-sf002.tif]
